# No Negative Impact of Palliative Sedation on Relatives’ Experience of the Dying Phase and Their Wellbeing after the Patient’s Death: An Observational Study

**DOI:** 10.1371/journal.pone.0149250

**Published:** 2016-02-12

**Authors:** S. M. Bruinsma, A. van der Heide, M. L. van der Lee, Y. Vergouwe, J. A. C. Rietjens

**Affiliations:** 1 Department of Public Health, Erasmus MC, Rotterdam, The Netherlands; 2 Helen Dowling Institute, Bilthoven, The Netherlands; Centre Hospitalier Universitaire Vaudois, FRANCE

## Abstract

**Background:**

Palliative sedation is the widely-used intervention of administering sedating agents to induce a state of unconsciousness to take away a dying patient’s perception of otherwise irrelievable symptoms. However, it remains questionable whether this ethically complex intervention is beneficial for patients and whether the associated lack of communication in the last phase of life has a negative impact on relatives’ wellbeing.

**Methods:**

An observational questionnaire study was conducted among relatives of a consecutive sample of patients who died a non-sudden death in the Erasmus MC Cancer Institute or in the hospice ‘Laurens Cadenza’ (both in Rotterdam) between 2010 and 2013.

**Results:**

Relatives filled in questionnaires regarding 151 patients who had been sedated and 90 patients who had not been sedated. The median time since all patients had passed away was 21 (IQR 14–32) months. No significant differences were found in relatives´ assessments of the quality of end-of-life care, patients´ quality of life in the last week before death and their quality of dying, between patients who did and did not receive sedation, or in relatives’ satisfaction with their own life, their general health and their mental wellbeing after the patient’s death.

**Conclusions:**

The use of sedation in these patients appears to have no negative effect on bereaved relatives’ evaluation of the patient’s dying phase, or on their own wellbeing after the patient’s death.

## Introduction

A frequently-used last resort intervention in end-of-life care is palliative sedation, i.e. the administration of sedating drugs to induce a state of unconsciousness to take away a dying patient’s perception of symptoms [1–3]. A European study undertaken in 6 countries in 2001/2 reported that continuous deep sedation until death was used for 2.5–8.5% of all dying patients [2]. Subsequent studies indicate that sedation was used for 15% of deceased persons in Flanders [Belgium] in 2007 [4], in 12% of all deceased persons in the Netherlands in 2010 [5], and in 17% of all deceased persons in the UK in 2007 [6,7]. Palliative sedation is used in all settings where patients die, but most often in hospitals and for patients with cancer [2,8–10]. While palliative sedation is often seen as an indispensable intervention to alleviate severe suffering in the dying phase, it is also heavily criticized for its resemblance to euthanasia [11], and for depriving patients of the ability to communicate when many of them strongly endorse the importance of being mentally aware [12]. Moreover, its use can be disturbing for the patient’s relatives [13,14]. For example, the start of sedation may be the moment at which the family becomes aware that the patient’s death is imminent and/or the intimacy of family care may be disrupted by the introduction of a medical technology, such as sedation [1].

Professionals working in palliative care stress the importance of good care for the relatives of the patient, because they are often present during the last period of the patient’s life and are closely involved with the patient. However, a literature review revealed that few studies have examined the experience of relatives with palliative sedation [15]; this review also showed that relatives’ experiences were mainly studied from the perspective of the professional caregivers. The authors conclude that most of the relatives had positive feelings regarding the use of palliative sedation, e.g. because palliative sedation helped to decrease the patient’s symptom distress. Finally, although most relatives reported to be comfortable with the use of sedation, some experienced the use of sedation as distressing [15].

The reasons for such distress include: i) the feeling that the patient still suffers while receiving sedation, ii) the inability to interact with the patient after the start of sedation, iii) feeling the burden of responsibility for the decision to start sedation, iv) concerns about a possibly hastened death, v) the (long) duration of the sedation, vi) the fact that information about the sedation cannot easily be obtained, and vii) the idea that there might be more appropriate ways to provide symptom relief [14–17]. As a result, sedation may negatively influence relatives’ experience of the patient’s dying phase, as well as their wellbeing after the patient’s death.

Insight into the potential consequences of the use of palliative sedation can support the development of evidence-based care strategies to improve the death experience for terminally ill patients and their relatives. Although most palliative care efforts focus on assessing and improving quality of life and quality of care for patients, many studies also emphasize the importance of taking good care of the patient’s relatives [18–20].

Therefore, this study examines whether the relatives of patients who receive palliative sedation differ in their experience of the dying phase and their wellbeing after the patient’s death compared to the relatives of patients who died a non-sudden death without the use of palliative sedation.

## Materials and Methods

### Study population and recruitment of participants

An observational study was conducted among relatives of a consecutive sample of 564 patients who had died a non-sudden death at the Erasmus MC Cancer Institute (a large academic hospital in the Netherlands with a specialized inpatient unit for palliative cancer care), or at the hospice ‘Laurens Cadenza’ (with 20 beds, the largest inpatient palliative care center in the Netherlands for patients for whom death is imminent) in Rotterdam between 2010 and 2013.

Relatives were not necessarily restricted to family members, but could also include significant others (e.g. close friends, etc.). Relatives who were registered as the contact person of the patient in the medical file were invited to take part in the study via an information letter sent by a senior clinical staff member of the two participating locations. If the relatives were willing to participate they were asked to fill in the questionnaire. If the relatives were not willing to participate they were asked to inform the research team via an answering sheet, or to ignore the invitation. When the relatives did not respond within six weeks, they received a reminder.

The study was approved by the Medical Ethical committee of the Erasmus MC (No. NL33327.078.10, v03). All participants gave written informed consent before participation in the study.

### Questionnaire

The questionnaire covered several topics shown to be important based on a systematic review on the experiences of relatives with palliative sedation [15]. Further, main themes relating to relatives’ experiences were identified in a focus group study among 14 relatives of patients who received palliative sedation until death [21] and in an interview study among 38 relatives of 32 cancer patients who received continuous sedation until death in three European countries [22]. The study questionnaire was based on several validated questionnaires [23–30]; all instruments have adequate psychometric properties [23,24,27,30–34].

To assess whether or not the patient had received sedation prior to death, relatives were asked the self-constructed question: ‘Has your relative been brought to sleep with medication prior to death?’ (yes/no/don’t know).

Relatives’ experience of the dying phase was assessed with three items that were derived from the Quality of Death and Dying questionnaire [23]: ratings on a 0–10 scale of 1) the patient’s quality of life in the last week before death, 2) their quality of dying, and 3) the quality of end-of-life care. On these scales, 0 represented a ‘terrible experience’ and 10 an ‘almost perfect experience’.

Relatives’ wellbeing after the patient’s death was assessed with several items. Relatives’ satisfaction with life 3 months after the patient’s death and at the time of the survey was measured with the Cantril Ladder [24], i.e. a picture of a ladder numbered from 0 on the bottom rung (worst possible life) to 10 on the top rung (best possible life). Relatives’ general health was assessed with a single item from the Short Form 36 Health Survey (SF-36), a self-report questionnaire [25] on which a score of 1 indicates poor health and a score of 5 excellent health. Mental wellbeing in the 4 weeks prior to the survey was assessed with the SF-36, which includes 5 items about mental health [25]; scores were linearly transformed from a 1–6 to a 0–5 scale. The sum score had a minimum of 0 and a maximum of 25. A low score indicates poor mental wellbeing (feelings of nervousness and depression all the time), and a high score implies excellent mental wellbeing (feels peaceful, happy, and calm all the time).

Questions on the sociodemographic characteristics of the deceased person and the relative were added by the research team [26,27]. These included questions about age, sex, relatives’ relationship to the patient (Married or partner/Child/ Other), highest level of education (Low = primary education + lower vocational education + lower secondary education; Intermediate = intermediate vocational education + upper secondary education; High = higher vocational education or university) and religion [Catholic/Christian/Other (e.g. Buddhism, Hinduism, Judaism)/No religion]. Items on relatives’ involvement in the care for the patient and the decision-making process, and the provision of information were based on the VOICES questionnaire [28]. Relatives were asked: 1) if they were involved in the care for the patient by the professional caregivers in the last week of life (Yes/No); 2) if they perceived the degree of involvement in care for the patient by the professional caregivers in the last week of life as sufficient (Yes/No, would have preferred more involvement/No, would have preferred less involvement); 3) if they perceived the amount of information received from professional caregivers about the situation of the patient and care for the patient during the last week of life as sufficient (Yes/No, received too much information/No, received too little information); 4) if they had the opportunity to say goodbye to the patient (Yes/More or less/No); 5) if they were present at the death of the patient (Yes/No, but another relative was present/No, and no other relatives were present); 6) if the professional caregivers could have done something to make the period before the death of the patient more bearable for the relative (Yes/No); and 7) if the professional caregivers could have done something for them to make the period after the death of the patient more bearable for the relative (Yes/No). The severity of the patient’s symptoms during the last week of life was assessed using items from the Edmonton Symptom Assessment System (ESAS) and the Structured Interview for Symptoms and Concerns (SISC) questionnaire [29,30]. The scale comprised 15 items, including 7 physical symptoms (i.e. pain, nausea/vomiting, dyspnea, confusion, restlessness, consciousness, fatigue) and 8 psychological symptoms (i.e. depression, anxiety, loss of control, loss of dignity, burden for environment, loss of interest, hopelessness, longing for death). Scores per item ranged from 1–5; the sum score had a minimum of 15 (not severe) and a maximum of 75 (very severe). The cause of death was recorded as ‘cancer’ or ‘other’ (cerebrovascular accident, respiratory diseases, dementia, heart failure and other diseases).

The content of the questionnaire was piloted among five respondents; this led to some minor changes in the formulation of the questions. In July and August 2013, 564 questionnaires were sent to potential participants.

### Data analyses

**Fig 1:**
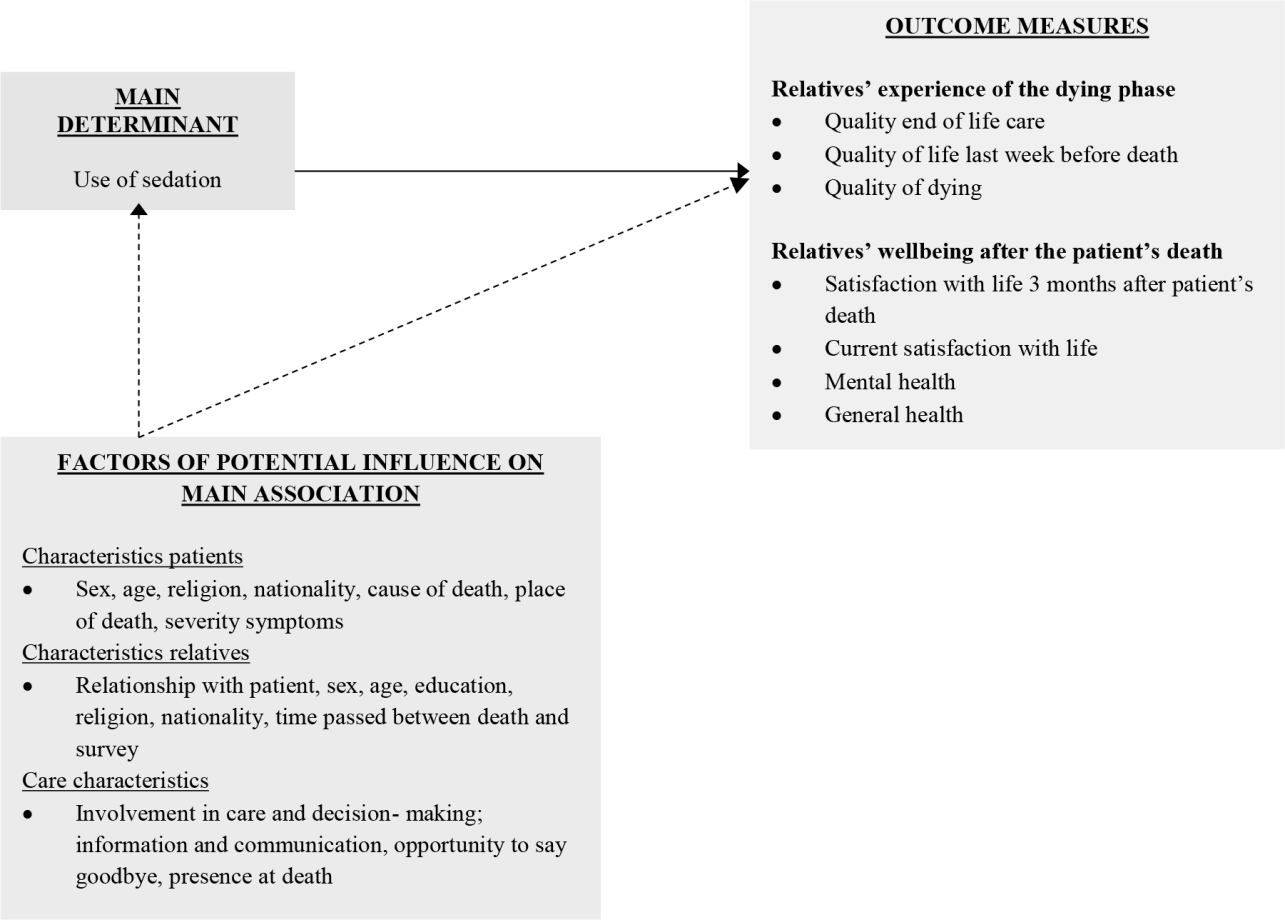
Model analyses.

Multivariable linear regression analysis was performed to assess whether the relatives of patients who received sedation differed in their experience of the dying phase and their wellbeing after the patient’s death from the relatives of patients who had died a non-sudden death without the use of palliative sedation. Because the error of the variance varied for several models (heteroscedasticity), robust estimates of standard errors and confidence intervals were assessed with bootstrapping [35]. To be consistent, this method was used for all models. Logistic regression was used to assess the association between the use of sedation (yes/no) and personal and care characteristics. Those variables that showed an association (p-value ≤ 0.30) were included in the main multivariable linear regression model (Fig 1).

## Results

**Fig 2:**
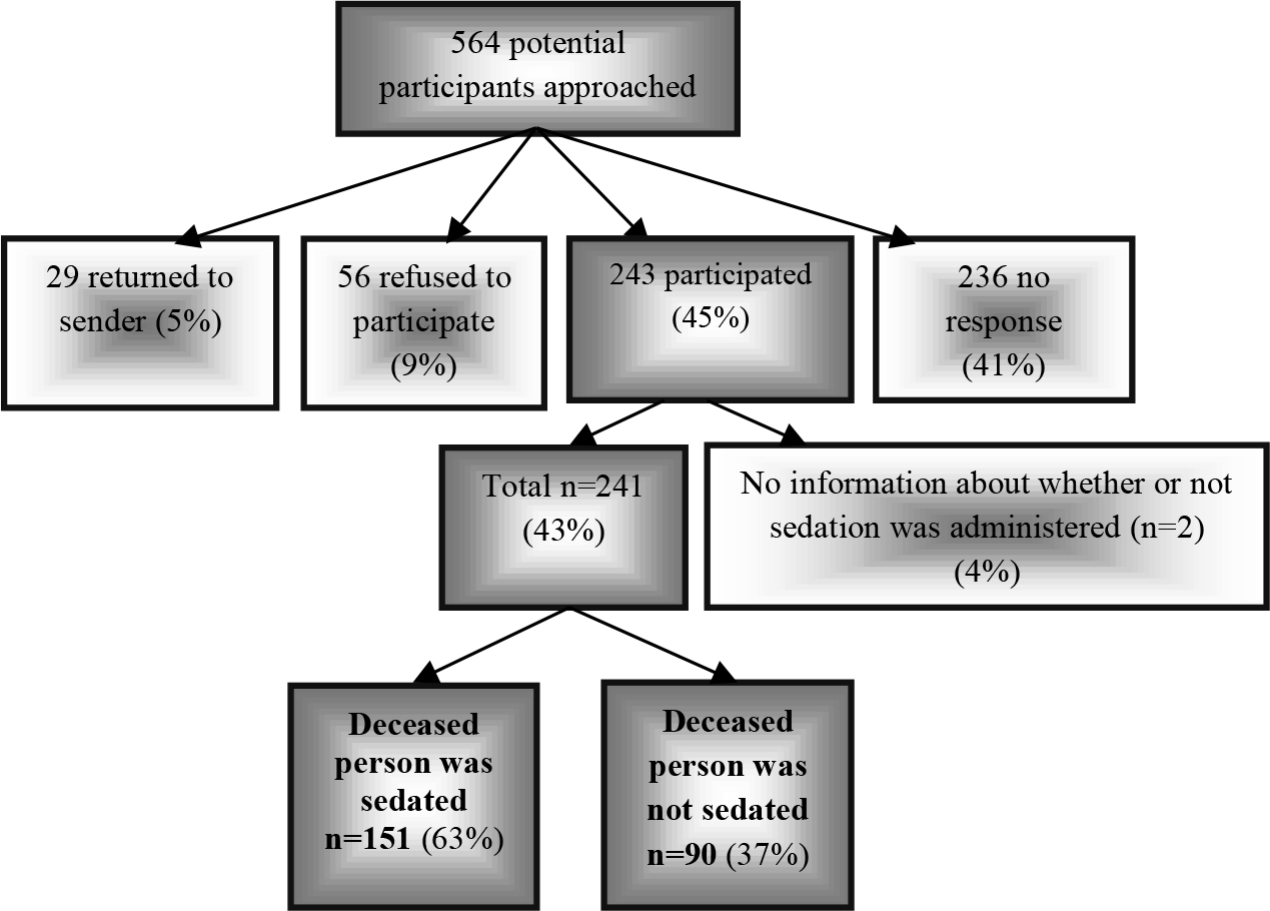
Flowchart of the inclusion of study participants.

Of the 564 relatives who were approached, 243 (45%) were willing to participate. Because two relatives did not answer the question on whether sedation had been administered 241 relatives (43%) were included, of which 151 (63%) concerned relatives of patients who had died after the use of palliative sedation. Fig 2 presents an overview of the inclusion of the relatives in the present study.

The characteristics of the deceased patients and their relatives are presented in Table 1. The median age at death of the non-sedated patients was slightly higher [77 (IQR 70–86) years] than that of the sedated patients [71 (IQR 62–81)] years (p = 0.002). Patients who received sedation prior to death more often died of cancer (92%) than patients who did not receive sedation (77%) (p = 0.002). Further, the median score for severity of symptoms during the last week of life was slightly higher (worse) for sedated patients [42 (IQR 34–52)] than for non-sedated patients [38 (IQR 30–46)] (p = 0.008). No significant differences were found with regard to patient’s sex, religion and place of death. Relatives of sedated patients were more often female (68%) than relatives of non-sedated patients (51%) (p = 0.007). No significant differences were found between both groups with regard to relatives’ relationship to the patient, age, education, religion, and time elapsed since bereavement.

**Table 1:** Characteristics of the deceased patients and their relatives (n = 241).

		No sedation (n = 90 patients)	Sedation (n = 151 patients)	p-value of difference^A^ Unadjusted
*Deceased patients*				
**Sex**	*Female*	50 (56%)	71 (47%)	0.187
	*Male*	39 (44%)	79 (53%)	
	*Missing*	1	1	
**Age in years** median (IQR) (n = 228)		77 (70–86)	71 (62–81)	0.002
**Religion**	*Catholic*	17 (22%)	22 (17%)	0.338
	*Christian*	21 (28%)	31 (23%)	
	*Other*^B^	6 (8%)	23 (17%)	
	*No religion*	32 (42%)	57 (43%)	
	*Missing*	14	18	
**Cause of death**	*Cancer*	62 (77%)	133 (92%)	0.002
	*Other*^C^	19 (23%)	12 (8%)	
	*Missing*	9	6	
**Place of death**	*Hospice*	83 (92%)	138 (91%)	0.821
	*Hospital*	7 (8%)	13 (9%)	
**Severity of symptoms during last week of life**^D^ *median (IQR)* (n = 201)	*Overall score*	38 (30–46)	42 (34–52)	0.008
*Relatives*				
**Relatives’ relationship to patient**	*Married/ partner*	24 (27%)	51 (36%)	.581
	*Child*	42 (48%)	54 (38%)	
	*Parent*	5 (6%)	12 (8%)	
	*Other*^E^	17 (19%)	26 (18%)	
	*Missing*	2	8	
**Sex**	*Male*	44 (49%)	47 (32%)	0.007
	*Female*	45 (51%)	101 (68%)	
	*Missing*	1	3	
**Age in years** median (IQR) (n = 237)		57 (49–66)	59 (50–65)	0.465
	*Missing*	1	3	
**Education**^F^	*Low*	37 (44%)	43 (31%)	0.400
	*Intermediate*	18 (21%)	55 (39%)	
	*High*	29 (35%)	43 (31%)	
	*Missing*	6	10	
**Religion**	*Catholic*	10 (13%)	15 (12%)	0.132
	*Protestant*	20 (25%)	33 (25%)	
	*Other*^B^	5 (6%)	19 (15%)	
	*No religion*	44 (56%)	63 (48%)	
	*Missing*	11	21	
**Time in months since bereavement** median (IQR) (n = 234)		20 (14–31)	22 (15–32)	0.592

^A^ Difference test is based on logistic regression analysis (univariable). Reference group is ‘no sedation’.

^B^ E.g. Buddhism, Hinduism, Judaism

^C^ cerebrovascular accident, respiratory diseases, dementia, heart failure and other diseases

^D^ Scale consisting of 15 items with range 1–5. Total scale score with a minimum of 15 (not severe) and a maximum of 75 (very severe). Symptoms: pain, nausea/vomiting, dyspnea, confusion, restlessness, depression, anxiety, consciousness, fatigue, loss of control, loss of dignity, burden for environment, loss of interest, hopelessness, longing for death.

^E^ Sibling, grandchild, cousin, family-in-law, aunt/uncle

^F^ Low = primary education + lower vocational education + lower secondary education; Intermediate = intermediate vocational education + upper secondary education; High = higher vocational education or university

Relatives of sedated patients more often stated that the professional caregivers could have done more to make the period before the death of the patient more bearable (n = 31, 20%) than relatives of non-sedated patients (n = 7, 8%) (p = 0.013). Such statements included suggestions to provide ‘more information’, e.g. regarding the drugs that were administered and the duration of the sedation, to provide ‘more compassioned care’, e.g. by being more attentive to relatives or offering a listening ear when needed, and general suggestions to provide ‘better care’. Otherwise, we found no significant differences between the groups in the characteristics of the care provided during the last week of life (Table 2). Relatives of non-sedated patients were involved in the care for the patient in 74% of the cases, and relatives of sedated patients in 83% of the cases (p = 0.093). Relatives perceived the degree of their involvement as sufficient in 84% and 88% of the cases, respectively (p = 0.357). For those relatives who perceived the degree of their involvement to be insufficient, all except one would have preferred more involvement. Relatives of non-sedated patients perceived the amount of information they received from professional caregivers about the patient’s situation and care during the last week of life as sufficient in 82% of the cases, compared to 88% of the relatives of sedated patients (p = 0.191). All relatives who did not perceive the amount of information as sufficient wished they had received more information. Relatives of non-sedated patients had had the opportunity to say goodbye to the patient in 53% of the cases and they had ‘more or less’ had the opportunity in 30%, versus 66% and 24% of relatives of sedated patients, respectively. Relatives of non-sedated patients were present at the patient’s death in 56%, compared to 60% of the relatives of sedated patients. In all cases where the relative was not present at the death of the patient, they indicated that another relative had been present. In total, 6% of the relatives of non-sedated patients stated that the professional caregivers could have done ‘something’ for them to make the period *after* the death of the patient more bearable, compared to 5% of the relatives of sedated patients (p = 0.700).

**Table 2:** Care characteristics (n = 241 relatives).

		No sedation (n = 90)	Sedation (n = 151)	Total	p-value ^a^
**Relative was involved in the care for the patient by the professional caregivers in the last week of life**	*Yes*	67 (74%)	126 (83%)	193 (80%)	.093
	*No*	23 (26%)	25 (17%)	48 (20%)	
**Relative perceived the degree of involvement in care for the patient by the professional caregivers in the last week of life as sufficient**	*Yes*	72 (84%)	132 (88%)	204 (86%)	.357
	*No*	14 (16%)	18 (12%)	32 (14%)	
	*Missing*	4	1	5	
**Relative perceived the amount of information received from professional caregivers about the patient’s situation and care during the last week of life as sufficient**	*Yes*	72 (82%)	132 (88%)	204 (86%)	.191
	*No*	16 (18%)	18 (12%)	34 (14%)	
	*Missing*	2	1		
Relative had the opportunity to say goodbye to the patient*	*Yes*	48 (53%)	98 (65%)	146 (61%)	.200
	*More or less*	27 (30%)	36 (24%)	63 (26%)	
	*No*	14 (16%)	15 (10%)	29 (12%)	
	*Missing*	1	2	3	
**Relative was present at the death of the patient**	*Yes*	50 (56%)	91 (60%)	141 (58%)	.473
	*No*	40 (44%)	60 (40%)	100 (42%)	
**Professional caregivers could have done something to make the period *before* the death of the patient more bearable for the relative**	*Yes*	7 (8%)	31 (20%)	38 (16%)	.013
	*No*	81 (92%)	120 (80%)	201 (84%)	
	*Missing*	2	-	2	
**Professional caregivers could have done something to make the period *after* the death of the patient more bearable for the relative**	*Yes*	5 (6%)	7 (5%)	12 (5%)	.700
	*No*	81 (94%)	143 (95%)	224 (95%)	
	*Missing*	4	1	5	

a. Difference test is based on logistic regression analysis (univariable). Reference group is ‘no sedation’.

* Variable was dichotomized for the logistic regression analysis

Table 3 presents relatives’ experience of the dying phase and relatives’ wellbeing after the patient’s death for non-sedated and sedated patients. We found no significant differences between both groups in univariable analyses and multivariable models that controlled for the characteristics of the patients, relatives and care. The median score for the quality of end-of-life care was 9 in both groups, and the median score for quality of life was 4 for non-sedated and 3 for sedated patients. The median score for quality of dying was 8 in both groups. Relatives’ satisfaction with their own life 3 months after the patient’s death was rated at a median of 6 in both groups; at the time of the survey the median scores were 8 and 7, respectively. The median scores on the general health scale were 3 in both groups, and the median scores on mental health were 11 and 12, respectively.

**Table 3:** Experience of the dying phase and wellbeing after the patient’s death: differences between relatives of patients who received no sedation and relatives of patients who died with the use of palliative sedation (n = 241).

	No sedation (n = 90)	Sedation (n = 151)	Univariable regression^A^		Multivariable regression^B^	
	*Median (IQR)*	*Median (IQR)*	*p-value*	*β (95% CI)*	*p-value*	*β (95% CI)*
***Relatives’ experience of the dying phase***						
**Quality end of life care**^**C**^ (n = 241)	9 (8–9)	9 (8–10)	0.808	0.06 (-0.43–0.52)	0.888	0.04 (-0.57–0.66)
**Quality of life of patient last week before death**^C^ (n = 241)	4 (2–7)	3 (2–7)	0.356	-0.35 (-1.12–0.30)	0.818	-0.13 (-1.21–0.96)
**Quality of dying**^C^ (n = 239)	8 (6–8)	8 (6–8)	0.324	0.31 (-0.32–0.99)	0.273	0.46 (-0.37–1.28)
***Relatives’ wellbeing after the patient’s death***						
**Life satisfaction 3 months after death of the patient**^C^ (n = 241)	6 (4–8)	6 (4–7)	0.082	-0.51 (-1.06–0.09)	0.970	0.01 (-0.74–0.77)
**Current life satisfaction**^C^ (n = 241)	8 (7–8)	7 (6–8)	0.268	-0.26 (-0.67–0.19)	0.581	-0.20 (-0.90–0.51)
**General health**^D^ (n = 236)	3 (3–4)	3 (3–3)	0.742	-0.39 (-2.69–1.92)	0.288	1.62 (-1.38–4.62)
**Mental health**^E^ (n = 232)	11 (9–14)	12 (12–14)	0.204	0.59 (-0.37–1.50)	0.371	0.61 (-0.73–1.95)

^A^ Linear regression (univariable)

^B^ Linear regression (multivariable). Adjusted for sex patient, age patient, cause of death, severity symptoms, religion relative, sex relative, involvement relative in care for patient, satisfaction relative with information from caregivers, opportunity to say goodbye to patient and care for relative before the death of the patient (p<0.30)

^C^ Range 0–10 (0 = terrible experience, 10 = almost perfect experience)

^D^ Range 1–5 (1 = poor health, 5 = excellent health)

^E^ Mental wellbeing 4 weeks before questionnaire. Scale with 5 items. The sum score had a minimum of 0 (low mental wellbeing) and a maximum of 25 (excellent mental wellbeing)

## Discussion

The aim of this study was to assess whether the relatives of patients who receive palliative sedation differ in their experience of the dying phase and their wellbeing after the patient’s death compared to the relatives of patients who died a non-sudden death without the use of palliative sedation. No significant differences were found in relatives´ assessments of the quality of end-of-life care, patients´ quality of life in the last week before death and their quality of dying. Further, no significant differences were found in relatives’ satisfaction with their own life, and their general health and their mental wellbeing after the patient’s death. It appears that the use of sedation may not have a negative effect on bereaved persons’ experience of the dying phase of their deceased relative or on their own wellbeing after the patient’s death, despite that relatives of sedated patients sometimes state that they lack support from professional caregivers before the death of the patient and that some relatives may experience the use of sedation as distressing [15,17,36].

Adequate symptom relief is a key to the experience of a ‘good’ death [12,37–39]. Many relatives evaluated the provision of palliative sedation to their severely suffering family member positively because the patient’s suffering is finally alleviated [21]. The benefit of palliation could explain why no differences were found in relatives’ experience of the dying phase and wellbeing after the patient’s death, despite the fact that relatives of sedated patients report significantly more severe symptoms in the last week of life than relatives of non-sedated patients; this has also been reported by others [9]. Further, the loss of the ability to communicate with the patient during the sedation, sometimes referred to as a ‘social death’ [40], and a potential life- shortening effect are often considered to be key drawbacks of the use of palliative sedation by healthcare professionals, ethical and legal experts [11,12]. However, in the evaluation of relatives, adequate symptom relief possibly outweighs these drawbacks. The wellbeing of the patient is known to be a crucial factor for the health and welfare of the patient’s relatives [41]. Therefore, adequate symptom relief for the patient might also benefit the wellbeing of relatives.

Providing information to family members and involving them in discussions about medical care and interventions is known to reduce symptoms of post-traumatic stress, anxiety, and depression [42,43]. Our study shows that most relatives experienced the amount of information they received from caregivers as sufficient, which might have been an important determinant of their wellbeing after the patient’s death. However, these findings are not fully in line with other studies that highlighted inadequate provision of information and poor communication in end-of-life care in general [19)], or with palliative sedation in particular [21,22]. One explanation for this could be that our study mainly concerned relatives of patients who were cared for in a hospice (91%), which is a place of care where bereaved family members have relatively few unmet needs regarding information [44].

The World Health Organization’s definition of palliative care incorporates a support system to help relatives to cope during the patient’s illness and during their own bereavement process [45]. Indeed, relatives need support for their own wellbeing as well as to enable them to be close to and support the patient [46]. In the present study, most relatives were satisfied with the support provided to them by the professional caregivers before and after the death of the patient. However, 1 in 5 relatives of sedated patients stated that the professional caregivers could have done something to make the period before the death of patient more bearable for them. Since palliative sedation is a far-reaching intervention that often follows a trajectory of intense suffering for the patient, caregivers should specifically focus on comforting, supporting and providing continuous information to the patient’s family when palliative sedation is being considered and while it is being administered.

The present study has several strengths and limitations. Since we conducted a study among relatives of consecutive patients, this implies that the senior clinical staff members could not have made a specific selection of patients or relatives; in addition, the large sample enhances the generalizability of our findings. On the other hand, non-response may have influenced our findings. For instance, persons with more severe feelings of grief are more prone to non-response than those with less feelings of grief [47,48]. Also, relatives may differ in their interpretation of the question ‘Has your relative been brought to sleep with medication prior to death’. Nevertheless, we think that this descriptive definition is less ambiguous than use of the term ‘palliative sedation’. Moreover, because lay persons have different notions about what this latter term entails [49], future studies should provide respondents with a clear definition of palliative sedation at the start of the study. Further, most relatives were recruited via a hospice and the quality of hospice care is generally rated higher than that of hospital care [50,51], also by relatives [28]. However, when we corrected the analysis for the place of death, the results remained unchanged. Therefore, regarding comparisons between the patient groups, the place of death is unlikely to have influenced the results. Also, heterogeneity within the investigated population (such as variability in the cause of death), may have lowered the power to find differences between the two patient groups. In the Netherlands, from 2002 onwards both euthanasia and physician-assisted suicide are legally allowed under certain conditions. It seems likely that legalizing euthanasia has an influence on both care practices and perceptions of what is important in end-of-life care. Thus, it should be noted that the present study was conducted within a specific context and that this might diminish the generalizability of our results to, for example, other countries. Finally, estimating the true effect of palliative sedation on relatives’ experiences of the dying phase and their wellbeing after the patient’s death would require an experimental study; however, this is of course not an option for this patient group.

In this study, although selection bias was probably limited, certain groups were not included in our sample, such as young caregivers and ethnic minorities (e.g. Turkish and Moroccan patients). Also, our study focused mainly on the experiences of relatives of cancer patients. Future studies should also include other patient populations, such as patients with dementia.

## Conclusions

Based on these findings, the use of sedation does not appear to have a negative influence on bereaved persons’ experience of the dying phase of their deceased relative or on their own wellbeing after the patient’s death. This finding might be attributed to relief of the patient’s severe suffering, or to relatives receiving sufficient information from their caregivers about palliative sedation. Nevertheless, palliative sedation is a far-reaching and ethically complex intervention that requires caregivers to focus on providing comfort, support and continuous information to both the patient and the patient’s family. Future research is necessary to gain a deeper understanding of the factors that contribute to the wellbeing of relatives prior to and after the patient’s death.
